# Heart Transplant Outcomes in Chemotherapy-Induced vs. Non-Ischemic-Dilated Cardiomyopathy: Pediatric and Adult Recipients in the Ventricular Assist Device Era

**DOI:** 10.31083/RCM48253

**Published:** 2026-04-24

**Authors:** Bibhuti B. Das, Seth Lirette, Swati Choudhry, Gopinath Perumal

**Affiliations:** ^1^Department of Pediatric Cardiology, Methodist Children’s Hospital, San Antonio, TX 78229, USA; ^2^Department of Data Science, University of Mississippi Medical Center, Jackson, MS 39216, USA; ^3^Department of Pediatric Cardiology, Texas Children’s Hospital, Baylor College of Medicine, Houston, TX 77030, USA; ^4^Department of Cardiovascular Surgery, University of Mississippi Medical Center, Jackson, MS 39216, USA

**Keywords:** cardio-oncology, chemotherapy-induced cardiomyopathy, heart transplantation, ventricular assist device, malignancy, Pediatric transplantation, non-ischemic cardiomyopathy

## Abstract

**Background:**

Chemotherapy-induced dilated cardiomyopathy (CIDCM) has become an increasingly recognized indication for heart transplantation (HT) among cancer survivors with end-stage heart failure (HF). Advances in cardio-oncology practices, mechanical circulatory support, and refined immunosuppression strategies have improved outcomes; however, comparative data with non-ischemic dilated cardiomyopathy (NIDCM) in the modern ventricular assist device (VAD) era remain limited. Therefore, this study primarily aimed to compare post-transplant outcomes between CIDCM and NIDCM within pediatric and adult cohorts in the VAD era.

**Methods:**

Data from the United Network for Organ Sharing (UNOS) registry were used to retrospectively analyze first-time orthotopic HT recipients between January 2010 and March 2023, with follow-up through March 2024. CIDCM was defined using the UNOS diagnosis codes “dilated myopathy-adriamycin” or “dilated myopathy-cancer”, whereas NIDCM included idiopathic, familial, myocarditis-related, and other specific DCM subtypes. Primary outcomes were post-HT survival, treated allograft rejection, and new or recurrent malignancy.

**Results:**

Among 28,813 recipients, 527 had CIDCM (52 pediatric, 475 adults). Pediatric survival was comparable between groups (1-, 5-, and 10-year survival: 0.92, 0.86, 0.76 vs. 0.95, 0.82, 0.68; *p* = 0.951). Adults with CIDCM showed superior survival (0.92, 0.82, and 0.68 vs. 0.91, 0.79, and 0.59; *p* = 0.018; hazard ratio (HR) 0.78 (0.64–0.96)) and lower rejection rates (0.03 vs. 0.04 events/person-year; *p* = 0.0027), with similar incidence of post-HT malignancy. Older age, female sex, and minority race were associated with reduced survival. In pediatric recipients, age >10 years and Ebstein Bar Virus (EBV) seronegativity were associated with post-HT malignancy; in adults, age ≥50 years was predictive.

**Conclusions:**

HT in CIDCM achieves durable survival and safety comparable to NIDCM. These results support expanding HT eligibility and multidisciplinary evaluation for cancer survivors with advanced HF in the contemporary era.

## 1. Introduction

As of 2025, an estimated 18.6 million cancer survivors live in the United States, representing roughly 5.5% of the population [1]. This growing cohort reflects major advances in oncologic therapies, but it also poses unique challenges for the management of advanced heart failure (HF) among long-term survivors of cancer [2]. Agents most commonly associated with chemotherapy-induced dilated cardiomyopathy (CIDCM) include anthracyclines (e.g., adriamycin), alkylating agents, immune checkpoint inhibitors, tyrosine kinase inhibitors, and human epidermal growth factor receptor (HER)2-targeted therapies (e.g., trastuzumab), particularly when used in combination with anthracyclines [3]. Topoisomerase 2 is considered a key mediator in anthracycline-induced cardiac toxicity [4]. In cardiomyocytes, the inhibition of topoisomerase 2 β results in breaks in DNA, leading to mitochondrial dysfunction, generation of reactive oxygen species, and activation of apoptotic pathways. The destruction of cardiomyocytes results in a progressive decrease in left ventricular (LV) function.

Childhood cancer survivors face particularly high cardiovascular risk, studies report up to a 15-fold increased risk of HF, and a 7-fold increased premature cardiac death compared to peers without such history [1]. Given the expanding population of cancer survivors with end-stage HF, advanced therapies such as ventricular assist devices (VAD) and heart transplantation (HT) must be considered for selected patients [5]. Prior studies in both pediatric and adult CIDCM suggest survival after transplantation is comparable to other cardiomyopathies [6, 7, 8]. However, concerns remain about recurrence of malignancy [9, 10, 11]. Many earlier reports grouped heterogeneous cardiomyopathy phenotypes including restrictive and hypertrophic types complicating outcome interpretation [9, 12]. To address these gaps, we analyzed the United Network for Organ Sharing (UNOS) registry data from 2010 onward period characterized by widespread VAD use and modern HF care and compared post-transplant mortality, rejection, and malignancy among pediatric and adult HT recipients with CIDCM versus those with non-ischemic dilated cardiomyopathy (NIDCM). The primary objective was to compare post-transplant outcomes between CIDCM and NIDCM within pediatric and adult cohorts, rather than to directly compare children versus adults. Our primary hypothesis was that HT outcomes in both pediatric and adult CIDCM patients would be suboptimal, and that a history of pre-transplant malignancy would be associated with an increased risk of post-transplant malignancy.

## 2. Methods

We conducted a retrospective cohort study using the UNOS registry, including patients who underwent first-time orthotopic HT for either CIDCM or NIDCM between January 1, 2010, and March 24, 2023. Follow-up data were available through March 2024. We categorized recipients as pediatric (<18 years at listing) or adult (≥18 years). Patients were classified into the CIDCM if their primary diagnosis field in the UNOS registry corresponded to “dilated myopathy – adriamycin” or “dilated myopathy – cancer”, which served as the primary cohort designation for both pediatric and adult populations. These diagnosis codes align with prior published UNOS analyses of Adriamycin-associated DCM [13]. Patients with NIDCM were identified based on a primary diagnosis of idiopathic, familial, myocarditis-related, or other specified subtypes of dilated cardiomyopathy (DCM); this group served as the comparison cohort in both pediatric and adult populations. We excluded patients whose diagnosis was congenital heart disease (CHD), ischemic cardiomyopathy, hypertrophic cardiomyopathy, restrictive cardiomyopathy, re-transplantation, or multiorgan transplantation (including heart + bone marrow). The de-identified UNOS data is available upon request for research analysis purposes, and does not require IRB or ethical approval, as all participating centers contributing to the registry have signed agreements permitting the sharing of anonymized data.

### 2.1 Rationale for Focusing on the Dilated Phenotype

We limited our CIDCM cohort to the dilated phenotype because: (1) DCM is the most common morphological manifestation of chemotherapy-induced cardiotoxicity and is frequently responsive to advanced HF therapies, including VAD support, a key focus of the present study; (2) other phenotypes such as restrictive physiology frequently seen after chest irradiation or in mixed cancer therapy exposures carry distinct pathophysiological characteristics and outcome trajectories [14], which would hamper direct comparison with the NIDCM cohort and not amenable to VAD support; (3) prior UNOS analyses of adriamycin-associated cardiomyopathy similarly excluded hypertrophic and restrictive forms [13]. However, the UNOS database lacks detailed oncologic variables such as cancer stage, specific chemotherapeutic agents, cumulative dosing, radiation exposure, and remission duration prior to transplantation. As a result, distinguishing among specific cardiotoxic mechanisms (e.g., anthracyclines, HER2-targeted therapies, immune checkpoint inhibitors) was not feasible, and some degree of misclassification may have occurred.

### 2.2 Data Extraction and Variables

We extracted demographic and clinical characteristics including age, sex, race/ethnicity, weight, body mass index (BMI), listing status, wait-list duration, prior oncologic diagnosis, Ebstein Bar Virus (EBV) and cytomegalovirus (CMV) serostatus, blood type, use of mechanical circulatory support (MCS) (including percutaneous and durable VADs), ventilatory/inotropic support, baseline hemodynamics at listing, induction immunosuppression at transplant and maintenance immunosuppression at discharge. Post-transplant outcomes were assessed using UNOS event and cause fields, specifically capturing instances of treated allograft rejection and de novo or recurrent malignancy.

### 2.3 Outcomes

Primary post-transplant outcomes included (1) post-transplant mortality, defined as time from transplant to death or graft failure requiring re-transplantation, censored at last follow-up; (2) incidence of treated allograft rejection; and (3) occurrence of new or relapsed malignancy following HT.

### 2.4 Statistical Analysis

Continuous variables were summarized as medians with interquartile ranges (IQRs) and compared using the Wilcoxon–Mann–Whitney test. Categorical variables were presented as counts and percentages and compared using the Chi-square test or Fisher’s exact test, as appropriate. Post-transplant survival, and freedom from treated rejection were analyzed using Kaplan–Meier (K–M) curves to depict post-transplant mortality and rejection-free probabilities. Group comparisons were assessed using log-rank tests and Cox proportional hazards models, with hazard ratios and 95% confidence intervals reported. Covariates for multivariable models were selected a priori based on established associations with post-transplant outcomes and clinical relevance, including demographic factors (age, sex, race/ethnicity), markers of illness severity at transplant (mechanical circulatory support and/or inotrope use), and transplant-related variables. Analyses employed complete-case methodology; missing data were not imputed. Select laboratory and serologic variables, such as EBV and CMV serostatus, had higher rates of missingness in the pediatric cohort and were included only in age-appropriate models when biologically relevant and sufficiently complete. Multivariable analyses were performed using Cox proportional hazards regression models to estimate adjusted hazard ratios (HRs) with corresponding 95% confidence intervals (CIs). All tests were two-tailed, and a *p*-value < 0.05 was considered statistically significant. Statistical analyses were performed using Stata version 19 (Stata Corp, College Station, TX, USA). Cause-specific mortality analyses were descriptive; competing-risks regression was not performed due to limited cause-specific event counts and the study’s primary focus on all-cause mortality. No formal adjustment for multiple comparisons was performed, as the primary analyses were prespecified and hypothesis-driven, focusing on clinically distinct outcomes (mortality, rejection, and malignancy). Results are therefore interpreted in the context of effect size, consistency across analyses, and biological plausibility.

## 3. Results

### 3.1 Study Population

**Supplementary Fig. 1** illustrates the CONSORT-style flow diagram of patient selection from the UNOS registry. After application of predefined inclusion and exclusion criteria, the final study population comprised 52 pediatric and 475 adult recipients with CIDCM, and 2240 pediatric and 26,046 adult recipients with NIDCM. Table 1 summarizes the baseline characteristics of the pediatric cohorts. Compared with NIDCM, CIDCM recipients were older (median 10.9 vs. 6.1 years; *p* < 0.001), more often female (66% vs. 49%; *p* = 0.04), and heavier (39.6 vs. 27.3 kg; *p* = 0.005). Ventilatory support was less common in CIDCM (26% vs. 46%; *p* = 0.018). Other listing characteristics, hemodynamics, and maintenance/induction immunosuppression were similar.

**Table 1.** **Comparative demographic, clinical, and hemodynamic profiles of pediatric CIDCM and NIDCM patients**.

Variable			NIDCM (N = 2240)	CIDCM (N = 52)	*p*-value
Demographics					
	Age, years, median (IQR)		6.11 (6.15)	10.89 (4.42)	<0.001
	Male sex, n (%)		852 (51)	18 (34)	0.044
	Weight, kg, median (IQR)		27.29 (26.97)	39.57 (19.49)	0.005
	Body mass index, kg/m^2^, median (IQR)		18.42 (5.03)	19.87 (4.70)	0.077
	Non-White race, n (%)		934 (56)	30 (58)	0.778
Transplant status					
	Status—urgent, n (%)		1506 (90)	46 (89)	0.855
	Status—somewhat urgent, n (%)		148 (9)	4 (8)	0.785
	Status—not urgent, n (%)		26 (2)	2 (3)	0.500
	Waitlist duration, days, median (IQR)		102.6 (197.1)	77.3 (99.9)	0.430
Infectious serostatus					
	EBV seropositive, n (%)		1785 (80)	29 (56)	0.002
	CMV seropositive, n (%)		1486 (66)	34 (65)	0.762
Blood type					
	A, n (%)		527 (31)	22 (42)	0.243
	B, n (%)		254 (15)	6 (11)	0.460
	AB, n (%)		65 (4)	4 (8)	0.227
	O, n (%)		834 (50)	20 (39)	0.132
Mechanical & medical support					
	Any VAD support, n (%)		985 (53)	23 (44)	0.940
		LVAD	732 (44)	19 (53)	0.340
		RVAD	2 (0)	0 (0)	1.00
		Total artificial heart	6 (0)	0 (0)	0.842
		LVAD + RVAD	134 (8)	4 (8)	1.00
	VAD type unknown		111 (11.2)	0 (0)	0.001
	ECMO, n (%)		49 (3)	0 (0)	0.285
	IABP, n (%)		8 (0)	0 (0)	0.670
	Inotropic support, n (%)		649 (39)	28 (47)	0.287
	Ventilatory support, n (%)		766 (46)	13 (26)	0.018
	Dialysis, n (%)		44 (3)	2 (3)	0.996
Laboratory values					
	Creatinine, mg/dL, median (IQR)		0.47 (0.51)	0.67 (0.37)	0.020
	Albumin, g/dL, median (IQR)		3.60 (0.76)	3.77 (0.91)	0.171
	Total bilirubin, mg/dL, median (IQR)		0.92 (2.63)	1.31 (1.40)	0.355
Hemodynamics					
	Cardiac output, L/min, median (IQR)		2.93 (1.73)	3.21 (1.17)	0.427
	PCWP, mmHg, median (IQR)		16.99 (8.28)	19.13 (6.52)	0.155
	Mean pulmonary artery pressure, mmHg, median (IQR)		25.97 (10.35)	28.91 (10.81)	0.116
Immunosuppression					
	Induction—antithymocyte globulin, n (%)		851 (38)	19 (36.5)	0.84
	Induction—IL-2 receptor antagonist, n (%)		717 (32)	18 (34.6)	0.77
	Induction—other, n (%)		89 (4)	1 (2)	0.18
Discharge immunosuppression					
	Calcineurin inhibitor, n (%)		2150 (96)	48 (92)	0.18
	Antimetabolite, n (%)		2217 (99)	51 (98)	0.63
	Corticosteroid, n (%)		2061 (92)	47 (91)	0.83

NIDCM, non-ischemic dilated cardiomyopathy; CIDCM, chemotherapy-induced dilated cardiomyopathy; IQR, inter quartile range; BMI, body mass index; EBV, Ebstein Bar Virus; CMV, cytomegalovirus; ECMO, extracorporeal membrane oxygenation; IABP, intra-aortic balloon pump; VAD, ventricular assist device; RVAD, right ventricle VAD; LVAD, left ventricle VAD; CO, cardiac output; PCWP, pulmonary capillary wedge pressure; mPAP, mean pulmonary artery pressure; IL, interleukin.

Baseline characteristics are summarized in Table 2 for the adult cohort. CIDCM recipients were younger (52.0 vs. 54.2 years; *p* < 0.001), predominantly female (74% vs. 24%; *p* < 0.001), with lower weight and BMI (both *p* < 0.001). Listing urgency, blood type, and use of inotropes or ventilatory support were comparable. For all outcomes, *p*-values were additionally adjusted using the Benjamini–Hochberg false discovery rate (FDR) procedure; results are presented in **Supplementary Table 1**. Key findings were consistent after FDR adjustment. Sensitivity analyses demonstrated consistent effect estimates across model specifications (**Supplementary Table 2**).

**Table 2.** **Comparative demographic, clinical, and hemodynamic profiles of adult CIDCM and NIDCM patients**.

Variable			NIDCM (N = 26,046)	CIDCM (N = 475)	*p*-value
Demographics					
	Age, years, median (IQR)		54.18 (12.25)	51.99 (12.76)	<0.001
	Male sex, n (%)		19,756 (76)	125 (26)	<0.001
	Weight, kg, median (IQR)		84.69 (18.21)	71.87 (15.58)	<0.001
	Body mass index, kg/m^2^, median (IQR)		27.84 (4.92)	25.91 (4.97)	<0.001
	Non-White race, n (%)		9790 (38)	172 (36)	0.539
Transplant status					0.143
	Status—urgent, n (%)		18,118 (70)	330 (69)	
	Status—somewhat urgent, n (%)		6801 (26)	116 (24)	
	Status—not urgent, n (%)		1127 (4)	29 (6)	
	Waitlist duration, days, median (IQR)		217.7 (379.3)	196.8 (353.4)	0.233
Infectious serostatus					
	EBV seropositive, n (%)		23,962 (92)	446 (94)	0.150
	CMV seropositive, n (%)		20,315 (78)	380 (80)	0.320
Blood type					0.243
	A, n (%)		10,283 (39)	184 (39)	
	B, n (%)		3954 (15)	65 (14)	
	AB, n (%)		1447 (6)	36 (8)	
	O, n (%)		10,362 (40)	190 (40)	
Mechanical & medical support					
	Any VAD support, n (%)		12,762 (49)	185 (39)	0.001
		LVAD	11,519 (44)	168 (35)	
		RVAD	56 (0)	0 (0)	
		Total artificial heart	208 (1)	1 (0)	
		LVAD + RVAD	523 (2)	11 (2)	
	VAD type unknown, n (%)		456 (3.6)	5 (1)	
	ECMO, n (%)		661 (3)	5 (1)	0.040
	IABP, n (%)		3869 (15)	73 (15)	0.755
	Inotropic support, n (%)		9912 (39)	181 (39)	0.939
	Ventilatory support, n (%)		4889 (19)	79 (17)	0.236
	Dialysis, n (%)		444 (2)	2 (0)	0.031
Laboratory values					
	Creatinine, mg/dL, median (IQR)		1.24 (0.56)	1.14 (0.55)	<0.001
	Albumin, g/dL, median (IQR)		3.70 (0.65)	3.70 (0.68)	0.903
	Total bilirubin, mg/dL, median (IQR)		0.96 (1.47)	1.08 (1.90)	0.101
Hemodynamics					
	Cardiac output, L/min, median (IQR)		4.53 (1.44)	4.05 (1.31)	<0.001
	PCWP, mmHg, median (IQR)		17.87 (8.96)	17.67 (8.42)	0.633
	Mean pulmonary artery pressure, mmHg, median (IQR)		27.22 (10.13)	27.10 (9.73)	0.814
Immunosuppression					
	Induction—antithymocyte globulin, n (%)		5730 (22)	100 (21)	0.65
	Induction—IL-2 receptor antagonist, n (%)		6771 (26)	128 (27)	0.63
	Induction—other, n (%)		520 (2)	9 (2)	0.87
Discharge immunosuppression					
	Calcineurin inhibitor, n (%)		24,795 (95.2)	448 (94.4)	0.45
	Antimetabolite, n (%)		25,030 (96.1)	458 (96.5)	0.65
	Corticosteroid, n (%)		24,535 (94.2)	442 (93.2)	0.32

NIDCM, non-ischemic dilated cardiomyopathy; CIDCM, chemotherapy-induced dilated cardiomyopathy; IQR, inter quartile range; BMI, body mass index; EBV, Ebstein Bar Virus; CMV, cytomegalovirus; ECMO, extracorporeal membrane oxygenation; IABP, intra-aortic balloon pump; VAD, ventricular assist device; RVAD, right ventricle VAD; LVAD, left ventricle VAD; CO, cardiac output; PCWP, pulmonary capillary wedge pressure; mPAP, mean pulmonary artery pressure; IL, interleukin.

### 3.2 Mechanical Circulatory Support

MCS utilization was categorized as durable VAD therapy or temporary support. Among adults, durable VAD implantation was significantly less frequent in patients with CIDCM compared to those with NIDCM (39% vs. 49%, *p* = 0.001). In the pediatric cohort, durable VAD use was comparable between CIDCM and NIDCM groups (approximately 44% vs. 53%, *p* = 0.940). Temporary percutaneous MCS with intra-aortic balloon pump (IABP) was used equally among adult CIDCM and NIDCM patients (15% each), whereas IABP use in pediatric patients was rare. Extracorporeal membrane oxygenation (ECMO) was less commonly employed in adults with CIDCM (1%) compared to NIDCM (3%, *p* = 0.040), and among pediatric patients, ECMO was not utilized in the CIDCM group but was used in 3% of those with NIDCM.

### 3.3 Post-transplant Survival

**Fig. 1.**
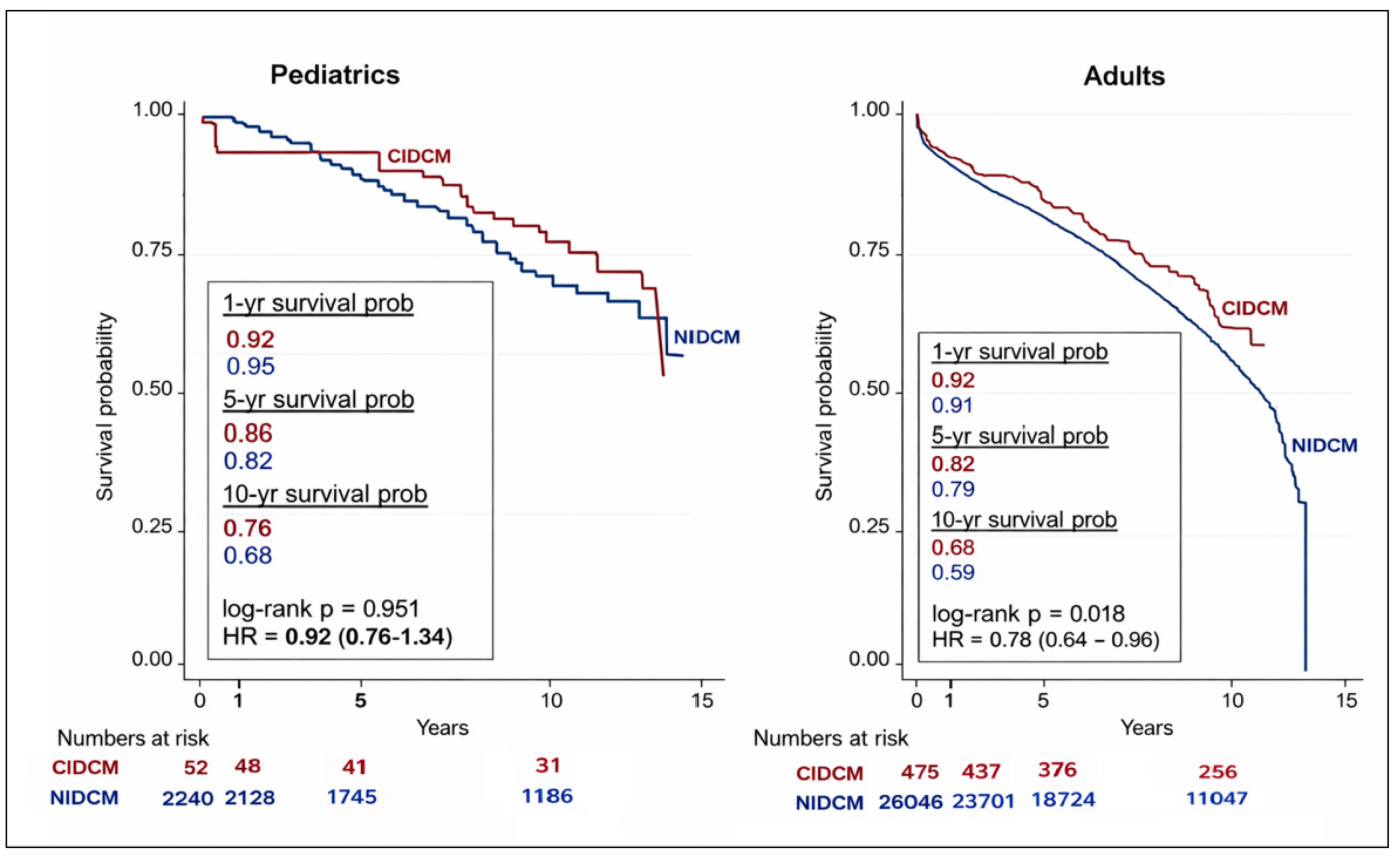
**Kaplan–Meier survival analysis of pediatric and adult heart transplant recipients with CIDCM and NIDCM**. NIDCM, non-ischemic dilated cardiomyopathy; CIDCM, chemotherapy-induced dilated cardiomyopathy.

Post-transplant survival outcomes are illustrated in Fig. 1. In the pediatric cohort, long-term survival did not differ significantly between patients with CIDCM and those with NIDCM, with 1-, 5-, and 10-year survival rates of 92%, 86%, and 76% for CIDCM versus 95%, 82%, and 68% for NIDCM (log-rank *p* = 0.951; hazard ratio [HR] 0.92, 95% confidence interval [CI] 0.76–1.34). In contrast, adult patients with CIDCM demonstrated significantly better survival compared to those with NIDCM, with respective 1-, 5-, and 10-year survival rates of 92%, 82%, and 68% versus 91%, 79%, and 59% (log-rank *p* = 0.018; HR 0.78, 95% CI 0.64–0.96).

**Fig. 2.**
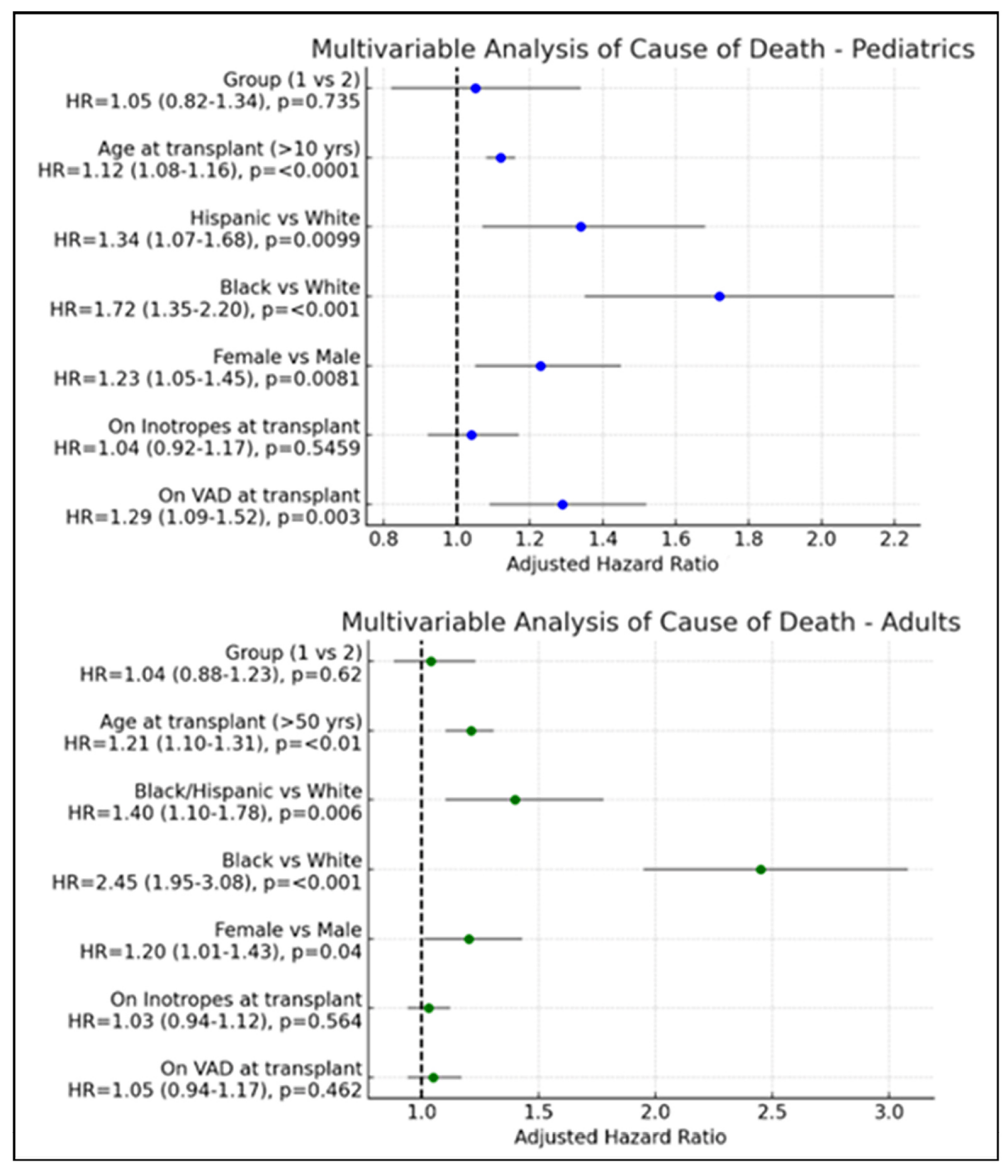
**Multivariable analysis of predictors of mortality**.

Multivariable Cox proportional hazards models evaluating all-cause mortality are shown in Fig. 2. In pediatric recipients, increased mortality risk was independently associated with age greater than 10 years at listing (HR 1.12 per year, *p* < 0.0001), female sex (HR 1.23, *p* = 0.0081), Black race (HR 1.72, *p* < 0.001), Hispanic ethnicity (HR 1.34, *p* = 0.0099), and use of a VAD at the time of transplant (HR 1.29, *p* = 0.003). Cardiomyopathy subtype (CIDCM vs. NIDCM) was not associated with mortality in pediatric recipients. Among adults, increased mortality risk was associated with age ≥50 years (HR 1.21, *p* < 0.01), female sex (HR 1.20, *p* = 0.04), and Black race (HR 2.45, *p* < 0.001). Pre-transplant inotrope use, VAD support, and cardiomyopathy subtype were not independently associated with mortality.

Cause-of-death distributions are summarized descriptively in Table 3A,3B. Overall, the relative proportions of death attributable to graft failure, infection, malignancy, and other causes were similar between cardiomyopathy subtypes. Among adult recipients, cerebrovascular deaths were significantly less frequent in the CIDCM group compared to those with NIDCM (12% vs. 21%, *p* = 0.0140), whereas no significant differences were observed in the pediatric cohort. These analyses were descriptive and not intended to estimate cumulative incidence in the presence of competing risks.

**Table 3A.** **Causes of post-HT deaths in pediatric recipients**.

Cause of death	CIDCM (n = 12)	NIDCM (n = 717)	*p*-value
Graft failure	4 (33.3%)	251 (35%)	1.000
Infection	2 (16.7%)	86 (12%)	0.640
Malignancy	2 (16.7%)	117 (16%)	1.000
Multiorgan failure	2 (16.7%)	150 (21%)	1.000
Encephalitis	1 (8.3%)	12 (2%)	0.266
Sudden death	1 (8.3%)	30 (4%)	0.444
Unknown	0 (0%)	71 (10%)	0.615

**Table 3B.** **Causes of post-HT deaths in adult recipients**.

Cause of death	CIDCM (n = 152)	NIDCM (n = 10,679)	*p*-value
Graft failure	20 (13%)	2029 (19%)	0.0851
Infection	33 (22%)	2349 (22%)	1.0000
Multiorgan failure	9 (6%)	405 (4%)	0.2518
Pulmonary	17 (11%)	961 (9%)	0.4290
Cerebrovascular	19 (12%)	2242 (21%)	0.0140
Hemorrhage	8 (5%)	427 (4%)	0.5616
Malignancy	6 (4%)	317 (3%)	0.6423
Unknown	40 (27%)	1949 (18%)	0.0145

NIDCM, non-ischemic dilated cardiomyopathy; CIDCM, chemotherapy-induced dilated cardiomyopathy.

### 3.4 Treated Rejections

**Fig. 3.**
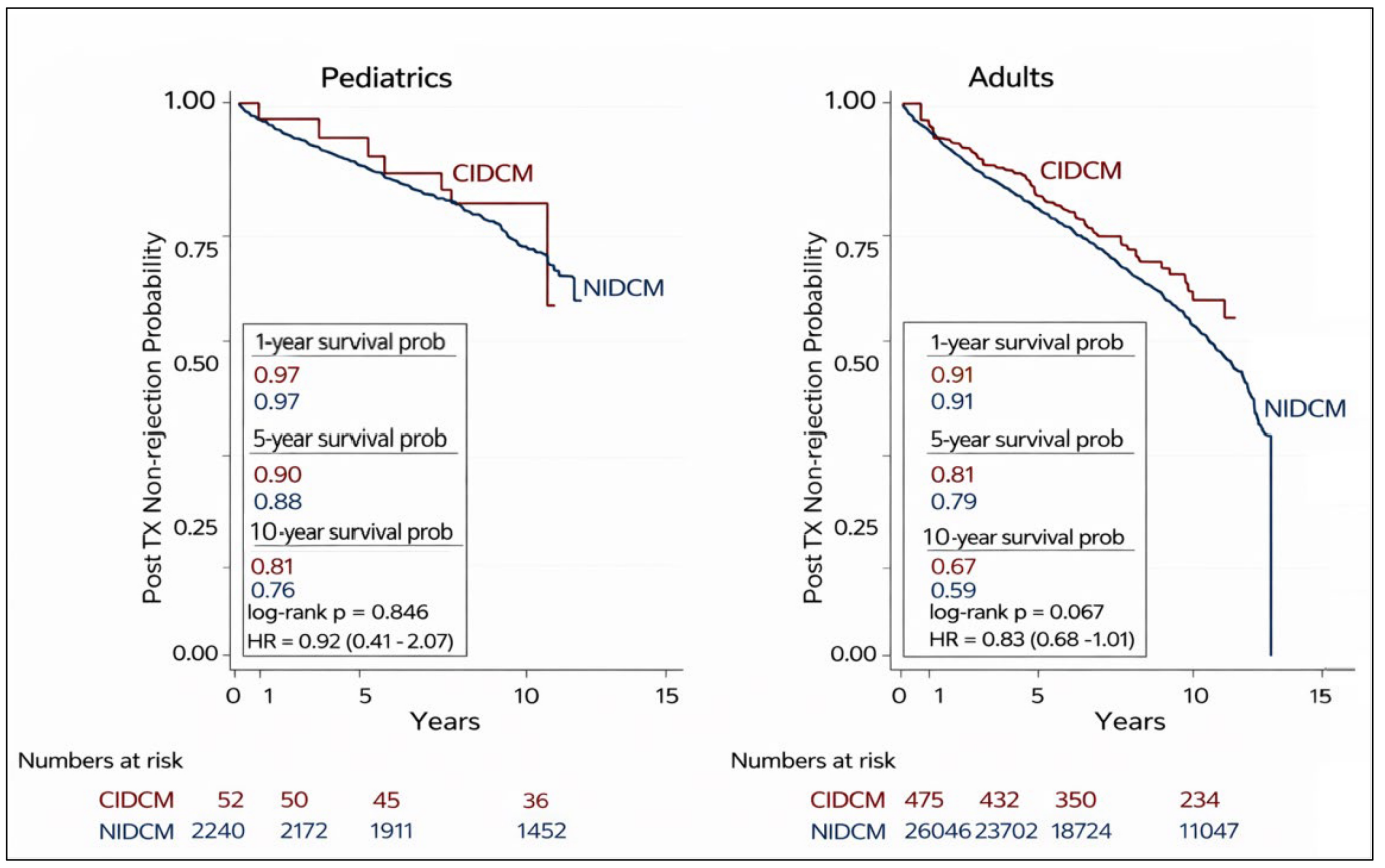
**Kaplan-Meier analysis of rejection free survival of pediatric and adult heart transplant recipients with CIDCM and NIDCM**. NIDCM, non-ischemic dilated cardiomyopathy; CIDCM, chemotherapy-induced dilated cardiomyopathy.

Treated rejection-free survival is illustrated in Fig. 3. In the pediatric cohort, rejection-free survival did not differ significantly between patients with CIDCM and those with NIDCM (log-rank *p* = 0.846; HR 0.92, 95% CI 0.41–2.07). Among adult transplant recipients, overall rejection-free survival was similar between groups, although a trend favoring improved outcomes in the CIDCM cohort was observed (log-rank *p* = 0.067; HR 0.83, 95% CI 0.68–1.01).

### 3.5 Malignancy

**Fig. 4.**
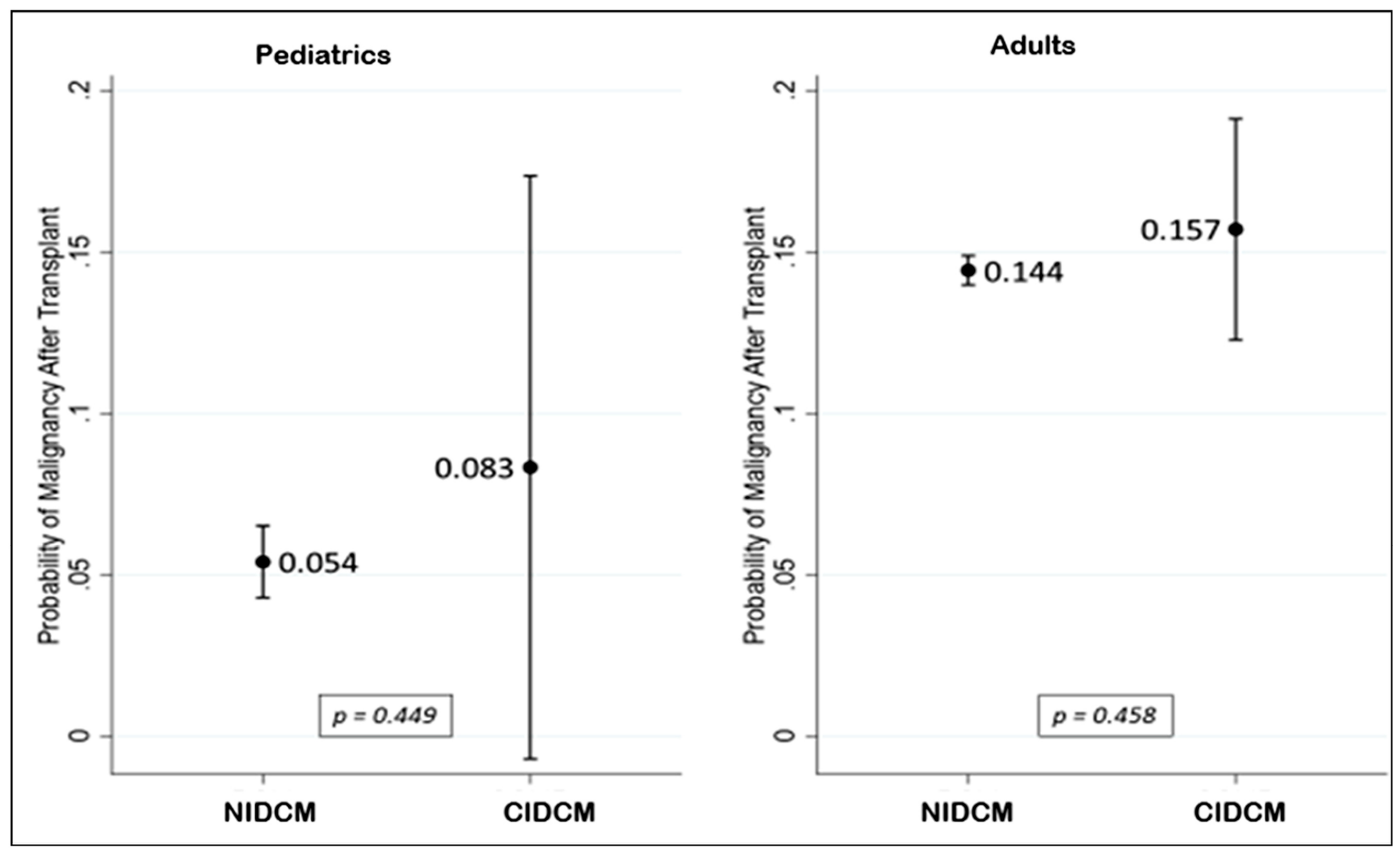
**Probability of post-transplant malignancy in pediatric and adult patients with CIDCM vs. NIDCM (hazard ratios with 95% confidence intervals derived from Cox proportional hazards models)**. NIDCM, non-ischemic dilated cardiomyopathy; CIDCM, chemotherapy-induced dilated cardiomyopathy.

**Fig. 5.**
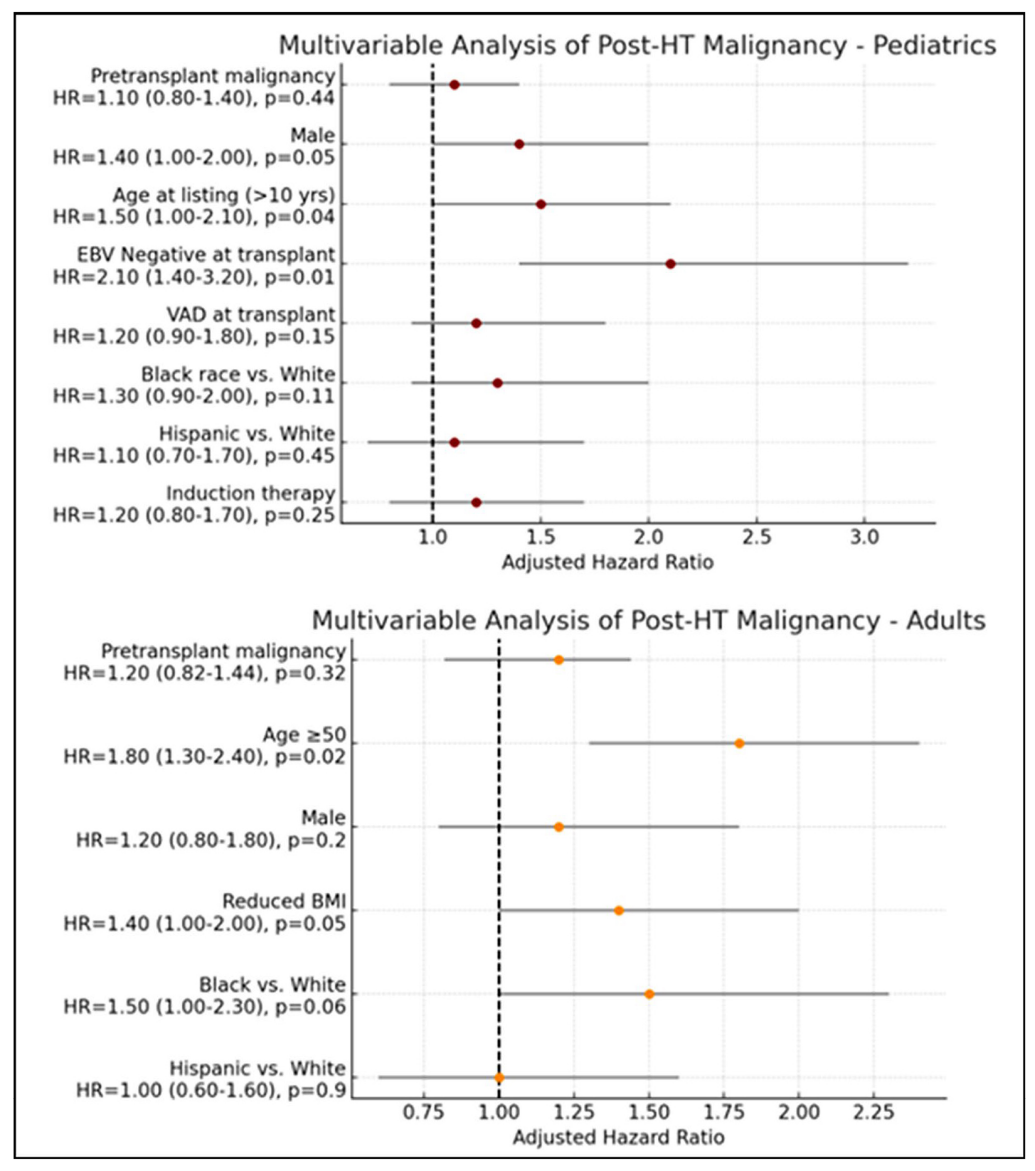
**Predictors of post-transplant malignancy in pediatric and adult patients with CIDCM**. CIDCM, chemotherapy-induced dilated cardiomyopathy; HT, heart transplantation.

The observed cumulative incidence of post-transplant malignancy was low and did not differ significantly between groups during available follow-up; however, these findings primarily reflect early- to mid-term outcomes. Among pediatric CIDCM recipients, the most frequently documented antecedent malignancies were leukemia (15%) and lymphoma (12%). In adult CIDCM recipients, prior breast cancer (42.5%) and leukemia (25.3%) were the predominant diagnoses (Table 4A,4B). Post-transplant malignancy rates are presented in Fig. 4. In the pediatric cohort, the incidence of malignancy was comparable between patients with CIDCM and those with NIDCM, occurring in 5.4% and 8.3% of cases, respectively (*p* = 0.449). Among adults, malignancy rates were also similarly distributed, with 14.4% in the CIDCM group and 15.7% in the NIDCM group (*p* = 0.458). Person-year analyses reflected consistent patterns (Table 5A,5B,5C). Post-HT malignancy types were detailed in Table 6A,6B. Among pediatric recipients with CIDCM, post-transplant malignancies were predominantly post-transplant lymphoproliferative disorder (PTLD) or of unknown histology. In contrast, adult CIDCM recipients developed most skin cancers, followed by PTLD and other solid organ malignancies. Risk factors for post-transplant malignancy are illustrated in Fig. 5. In the pediatric cohort, age greater than 10 years at the time of listing was associated with a significantly increased risk of post-transplant malignancy (HR 1.50, *p* = 0.04), as was EBV seronegativity at the time of transplant (HR 2.10, *p* = 0.01). No other covariates reached statistical significance in this group. Among adult recipients, age ≥50 years was independently associated with elevated malignancy risk (HR 1.80, *p* = 0.02), while pre-transplant malignancy history, sex, and race did not demonstrate significant associations.

**Table 4A.** **Pre-HT cancer diagnoses in pediatric recipients**.

Cancer type	CIDCM (N = 52)
Leukemia	8
Lymphoma	6
Oropharyngeal	1
Brain tumor	1
Osteosarcoma	1
Unknown	35

**Table 4B.** **Pre-HT cancer diagnoses in adult recipients**.

Cancer type		CIDCM (N = 475)
Single primary site		
	Breast	202 (42.5%)
	Leukemia	120 (25.3%)
	Skin melanoma	1 (0.2%)
	Skin non-melanoma	1 (0.2%)
	Genitourinary tract	3 (0.6%)
	Liver	2 (0.4%)
Multiple primary sites		
	Skin melanoma + Breast	2 (0.4%)
	Skin non-melanoma + Breast	2 (0.4%)
	Genitourinary tract + Breast	1 (0.2%)
	Breast + Thyroid	2 (0.4%)
	Breast + Lung	1 (0.2%)
	Skin non-melanoma + Leukemia	1 (0.2%)
	Brain + Leukemia	1 (0.2%)
	Genitourinary tract + Leukemia	1 (0.2%)
	Breast + Leukemia	6 (1.3%)
	Skin non-melanoma + Other	1 (0.2%)
	Genitourinary tract + Other	1 (0.2%)
	Breast + Other	8 (1.7%)
	Thyroid + Lung + Other	1 (0.2%)
	Leukemia + Other	3 (0.6%)
Unknown/Not specified		115 (24.2%)

**Table 5A.** **Comparative 10-year post–transplant mortality rates per 100 person-years in pediatric and adult recipients with CIDCM vs. NIDCM**.

Group	N	10-year survival	Estimated deaths	Deaths per 100 person-years	*p*-value
Pediatrics, CIDCM	52	0.76	12	2.4	0.246
Pediatrics, NIDCM	2240	0.68	717	3.2	
Adults, CIDCM	475	0.68	152	3.2	0.0006
Adults, NIDCM	26,046	0.59	10,679	4.1	

**Table 5B.** **Comparative 10-year post–transplant treated rejection rates per 100 person-years in pediatric and adult recipients with CIDCM vs. NIDCM**.

Group	N	10-year rejection-free survival	Estimated rejections	Rejections per 100 person-years	*p*-value
Pediatrics, CIDCM	52	0.81	10	1.9	0.624
Pediatrics, NIDCM	2240	0.78	493	2.2	
Adults, CIDCM	475	0.67	157	3.3	0.0027
Adults, NIDCM	26,046	0.59	10,679	4.1	

**Table 5C.** **Comparative 10-year post–transplant malignancy rates per 100 person-years in pediatric and adult recipients with CIDCM vs. NIDCM**.

Group	N	10-year cancer-free survival	Estimated malignancies	Malignancies per 100 person-years	*p*-value
Pediatrics, CIDCM	52	0.917	4	0.83	0.471
Pediatrics, NIDCM	2240	0.946	121	0.54	
Adults, CIDCM	475	0.845	74	1.55	0.583
Adults, NIDCM	26,046	0.855	3777	1.45	

NIDCM, non-ischemic dilated cardiomyopathy; CIDCM, chemotherapy-induced dilated cardiomyopathy; HT, heart transplantation.

**Table 6A.** **Spectrum of post–HT malignancies in pediatric recipients**.

Type of post-transplant malignancies	Estimated number	Percentage (%)
Recurrences (leukemia)	1	3.6%
Solid tumor	2	7.1%
Lymphoma	2	7.1%
PTLD	9	32.2%
Unknown	14	50%
Total	28	100%

**Table 6B.** **Spectrum of post–HT malignancies in adult recipients**.

Type of post-transplant malignancies	Estimated number	Percentage (%)
Skin cancer	32	47.1%
Lung cancer	2	2.9%
Prostate cancer	2	2.9%
PTLD	6	8.9%
Breast cancer	4	5.9%
Colorectal cancer	4	5.9%
Leukemia	2	2.9%
Esophageal cancer	2	2.9%
Recurrences of cancer (breast cancer)	1	1.5%
Unknown	13	19.1%
Total	68	100%

HT, heart transplantation; PTLD, post-transplant lymphoproliferative disorder.

## 4. Discussion

This large, contemporary UNOS registry analysis demonstrates that HT outcomes for patients with CIDCM due to anthracyclines are favorable and, in many respects, comparable to those with NIDCM. Adult CIDCM recipients exhibited superior long-term survival and a trend toward lower rejection rates, along with reduced cerebrovascular mortality. In contrast, pediatric outcomes including overall survival, rejection-free survival, and post-transplant malignancy rates were similar between CIDCM and NIDCM groups. Across both age cohorts, the incidence and types of post-transplant malignancy did not differ significantly by cardiomyopathy subtype. These findings support the premise that cancer survivors with advanced HF due to CIDCM phenotype can achieve durable post-transplant outcomes equivalent to, and in some cases exceeding, those of NIDCM candidates. The observed survival advantage among adult CIDCM recipients may also be influenced by residual confounding. In our cohort, adult CIDCM recipients were younger, and had lower BMI than NIDCM recipient factors associated with improved post-transplant outcomes. Although multivariable models adjusted for key demographic and transplant-related covariates, residual confounding from unmeasured factors such as comorbidity burden, frailty, cancer-related health status, and center-level practices cannot be excluded. Therefore, these findings should be interpreted as associative rather than causal.

### 4.1 Evolving Landscape of Management of Advanced HF in CIDCM 

Historically, cancer-related cardiomyopathy was associated with higher post-transplant risk due to concerns regarding recurrence, infection, and restrictive physiology [15]. Our findings, limited to the dilated phenotype and anthracycline chemotherapy, more accurately reflect patients who progress through contemporary VAD and HT pathways [16, 17, 18]. Improved outcomes likely result from advances in oncologic therapy, risk-based HF surveillance, and refined immunosuppression [19, 20]. The European Society of Cardiology (ESC) Cardio-Oncology Guidelines emphasize the role of multidisciplinary cardio-oncology teams, early HF detection, and careful surveillance in survivors receiving cardiotoxic therapy [21]. The International Society for Heart and Lung Transplantation (ISHLT) recommends individualized risk assessment in candidates with a history of malignancy, in collaboration with oncology specialists, to evaluate cancer-related survival and recurrence risk in the context of immunosuppression [17]. The Heart Failure Society of America (HFSA) statement similarly highlights integrated cardio-oncology care and the expanding role of advanced HF therapies for cancer survivors [5]. These guideline-driven shifts may have facilitated safer transplantation practices in this population. Durable VAD and ECMO use were lower in the CIDCM cohort compared to NIDCM, potentially reflecting earlier referral or more favorable pre-transplant status. This contrasts with earlier INTERMACS data (2006–2011), which reported higher rates of biventricular VAD use among CIDCM patients, likely due to a greater burden of right ventricular dysfunction [22]. However, in our analysis, we did not observe increased use RVADs in the CIDCM population. More recent analyses suggest similar outcomes between CIDCM and matched DCM cohorts, potentially reflecting improvements in management and risk assessment [23]. In alignment with the 2023 ISHLT guidelines, patients with a history of treated malignancy who are in sustained remission or considered disease-free may be eligible for VAD support either as destination therapy or as a bridge to transplant following comprehensive oncologic evaluation to assess recurrence risk and disease trajectory [16]. Differences in pre-transplant MCS utilization should be interpreted cautiously. Although adult recipients with CIDCM had lower rates of durable VAD support at transplantation, the UNOS registry does not capture referral timing, HF trajectory, or center-specific listing practices. As such, we cannot directly determine whether lower VAD utilization reflects earlier referral, differences in clinical severity, or provider decision-making. Observed differences may instead represent treatment-channeling and selection effects inherent to observational registry data rather than causal relationships.

Confounding by indication should be considered when interpreting differences in pre-transplant support strategies between CIDCM and NIDCM recipients. Patients with CIDCM are often younger, more frequently female, and may be listed earlier during advanced HF due to close oncology–cardiology follow-up and heightened surveillance, potentially reducing the need for bridging support. Conversely, clinicians may be more conservative with durable VAD implantation in cancer survivors because of perceived risks related to prior chemotherapy exposure (e.g., cytopenias), infection, bleeding, frailty, or uncertainty regarding cancer recurrence—factors that are not fully captured in UNOS. In addition, CIDCM can be accompanied by treatment-related comorbidities (e.g., renal dysfunction, pulmonary toxicity) that influence both listing urgency and device selection. Although we adjusted for VAD use and other key covariates in multivariable models, residual confounding related to unmeasured clinical factors and center-level practice variation cannot be excluded.

### 4.2 Post-Transplant Malignancy

The comparable rates of post-HT malignancy between CIDCM and NIDCM groups are reassuring, suggesting that prior anthracycline chemotherapy exposure does not independently increase malignancy risk in modern practice. Oncogenesis after HT remains multifactorial, driven by immunosuppression intensity, duration, viral co-infections (EBV, CMV), and age-related susceptibility [20]. In our pediatric cohort, EBV seronegativity and age >10 years independently predicted post-transplant malignancy, consistent with established PTLD risk profiles [24, 25]. In adults, only age ≥50 years was predictive. Our findings, consistent with prior studies [26, 27], reinforce AHA and ISHLT recommendations favoring tailored, risk-based post-transplant surveillance rather than categorical exclusion of patients with a history of malignancy, while underscoring the need for improved early detection strategies [5, 17].

### 4.3 Sex- and Age-Specific Considerations

Women comprised 74% of adult CIDCM recipients, with breast cancer as the leading antecedent diagnosis—consistent with prior reports identifying breast cancer as the most common malignancy associated with chemotherapy-induced cardiomyopathy [28]. Up to 7.6% of breast cancer survivors exposed to anthracyclines or HER2-targeted agents develop HF, most often among young adults who remain candidates for advanced therapies [29]. In this context, HT represents a potential treatment for end-stage HF, although long-term oncologic and graft outcomes require further study. In contrast, among pediatric transplant recipients, female sex has been independently associated with worse post-transplant survival. Prior studies across both congenital and acquired heart diseases have similarly reported poorer outcomes among adolescent females, potentially reflecting a multifactorial interplay of higher rates of medication nonadherence, illness-related anxiety, and psychosocial stressors that impair self-management and complicate transitions of care [30, 31]. Although the UNOS registry lacks data on adherence, psychosocial variables, and hormonal influences, existing literature suggests that adolescent females may face greater mental health burdens and transition-related challenges, which could contribute to adverse outcomes [32]. These divergent findings between adults and children likely reflect age-specific psychosocial and developmental factors rather than intrinsic biological risk, underscoring the need for prospective, mechanistic studies to clarify sex-based vulnerabilities across the lifespan.

### 4.4 Clinical Implications and Equity in HT Access

Our study demonstrated safety and efficacy of HT in CIDCM underscore the need to reconsider historical barriers to transplantation in cancer survivors. The ISHLT consensus statement now endorses individualized listing decisions, advocating multidisciplinary collaboration to assess recurrence risk and disease-free intervals rather than a fixed five-year remission requirement [17]. This approach promotes equitable access to HT and aligns with observed outcomes in our CIDCM cohort, in whom oncologic remission duration, younger age, and favorable health status likely contributed to superior survival and lower rejection rates.

## 5. Limitations

This study has several limitations inherent to its retrospective registry-based design. Selection bias and treatment-channeling effects are important considerations, as patients with CIDCM referred for HT likely represent a healthier subset of cancer survivors who completed therapy, achieved remission, and were deemed suitable for advanced HF interventions through multidisciplinary evaluation. Consequently, CIDCM recipients may differ systematically from NIDCM recipients in ways not fully captured by registry variables, potentially influencing observed outcome differences. Analyses involving pre-transplant MCS are subject to immortal time bias, as patients must survive long enough to receive durable VAD support and subsequently undergo transplantation. Although VAD use was modeled as a baseline covariate with adjustment for key clinical factors, residual confounding related to timing and duration of support cannot be excluded. Limited follow-up among patients transplanted between 2020 and 2023 may result in underrepresentation of late post-transplant events such as chronic rejection and de novo malignancy, particularly in the most recent era. While this study reflects a contemporary transplant landscape characterized by broader access to MCS and improved oncologic care, these advances complicate direct comparisons with earlier registry cohorts. Finally, as with all observational studies evaluating multiple outcomes, the absence of formal correction for multiple comparisons raises the possibility of type I error. Given the small size of certain subgroups, especially pediatric CIDCM recipients, these analyses should be considered exploratory, with emphasis on effect sizes and confidence intervals rather than statistical significance. The generalizability of findings is limited to chemotherapy-associated cardiomyopathy phenotypes identifiable through UNOS diagnostic coding, which predominantly reflects anthracycline-related DCM. Cardiomyopathies associated with newer oncologic agents—such as immune checkpoint inhibitors, tyrosine kinase inhibitors, and other targeted therapies—could not be specifically evaluated and may have distinct trajectories. Despite efforts to isolate CIDCM cases, misclassification with other NIDCM etiologies remains possible, particularly in adults, due to limited diagnostic granularity.

## 6. Conclusions

In this contemporary registry analysis, HT for CIDCM was associated with long-term survival and post-transplant outcomes that appeared comparable to, and in adults potentially more favorable than, those observed in recipients with NIDCM. Among adults, trends toward improved long-term survival, lower rejection rates, and no evident increase in post-transplant malignancy risk were observed. Pediatric outcomes appeared similarly reassuring, with no clear differences in survival or adverse events compared with NIDCM recipients. These findings suggest that HT can be a viable option for carefully selected cancer survivors with end-stage HF. Broader inclusion of CIDCM patients, supported by multidisciplinary evaluation, individualized immunosuppression, and sustained oncologic surveillance, may help advance equity and improve outcomes in this growing population. Alignment with current ESC, HFSA, and ISHLT cardio-oncology guidance will be essential to optimize transplant candidacy, mitigate recurrence risk, and support long-term survivorship in patients with cancer-related cardiomyopathy.

## Data Availability

The data are publicly available from the UNOS database upon application.

## Abbreviations

CIDCM, chemotherapy induced dilated cardiomyopathy; NIDCM, non-ischemic dilated cardiomyopathy; HR, hazard ratio; CI, confidence interval; IQR, inter quartile range; HT, heart transplantation; UNOS, United Network for Organ Sharing; RVAD, right ventricular assist device; LVAD, left ventricular assist device; TAH, total artificial heart; ESC, European Society of Cardiology; HFSA, Heart Failure Society of America; INTERMACS, Interagency Registry for Mechanically Assisted Circulatory Support.

## Supplementary Material

Supplementary material associated with this article can be found, in the online version, at https://doi.org/10.31083/RCM48253.
