# Supplementary material for: Heart Transplant Outcomes in Chemotherapy-Induced vs. Non-Ischemic-Dilated Cardiomyopathy: Pediatric and Adult Recipients in the Ventricular Assist Device Era
Source: Rev Cardiovasc Med. 2026 Apr 24;27(4):48253. doi: 10.31083/RCM48253 (PMC13155986; doi:10.31083/RCM48253)
Supplement: Supplementary file 1 [file 2153-8174-27-4-48253-s1.zip › Supplementary Material.docx]

**Supplementary Table 1.** Raw and FDR-Adjusted p-Values for Key Study Outcomes

| **Outcome** | **Raw p-value** | **FDR-adjusted p-value** |
| --- | --- | --- |
| Pediatric overall survival (log-rank) | 0.951 | 0.951 |
| Adult overall survival (log-rank) | 0.018 | 0.063 |
| Pediatric treated rejection | 0.846 | 0.951 |
| Adult treated rejection | 0.067 | 0.156 |
| Pediatric post-transplant malignancy | 0.449 | 0.641 |
| Adult post-transplant malignancy | 0.458 | 0.641 |
| Adult cerebrovascular cause of death | 0.014 | 0.063 |

(Raw p-values are derived from log-rank tests or χ²/Fisher’s exact tests, as appropriate. False discovery rate (FDR)–adjusted p-values were calculated using the Benjamini–Hochberg procedure to account for multiple comparisons across secondary outcomes. FDR-adjusted values are reported for transparency; primary inferences are based on prespecified outcomes.)

**Supplementary Table 2. Sensitivity Analyses for Multivariable Survival Models**

**Pediatric recipients**

| Model Specification | Covariates Included | Adjusted HR for CIDCM vs. NIDCM (95% CI) | p-value |
| --- | --- | --- | --- |
| Primary multivariable model | Age at transplant, sex, race/ethnicity, VAD use, EBV serostatus | 0.92 (0.76–1.34) | 0.735 |
| Complete-case analysis | Same as primary model, excluding missing EBV/CMV data | 0.94 (0.78–1.36) | 0.781 |
| Excluding EBV serostatus | Age, sex, race/ethnicity, VAD use | 0.93 (0.77–1.35) | 0.748 |

**Adult Recipients**

| Model Specification | Covariates Included | Adjusted HR for CIDCM vs. NIDCM (95% CI) | p-value |
| --- | --- | --- | --- |
| Primary multivariable model | Age, sex, race/ethnicity, BMI, VAD use at transplant, transplant era | 0.78 (0.64–0.96) | 0.018 |
| Complete-case analysis | Same as primary model, excluding cases with missing covariates | 0.80 (0.66–0.98) | 0.026 |
| Excluding EBV serostatus | Age, sex, race/ethnicity, BMI, VAD use, transplant era | 0.79 (0.65–0.97) | 0.021 |

(Sensitivity analyses were performed to assess the impact of missing data on multivariable survival models. Complete case analyses excluded recipients with missing covariates. Additional models excluded Epstein–Barr virus (EBV) serostatus to evaluate robustness given higher missingness in pediatric recipients. Effect estimates were consistent across models, supporting the stability of the primary findings.)


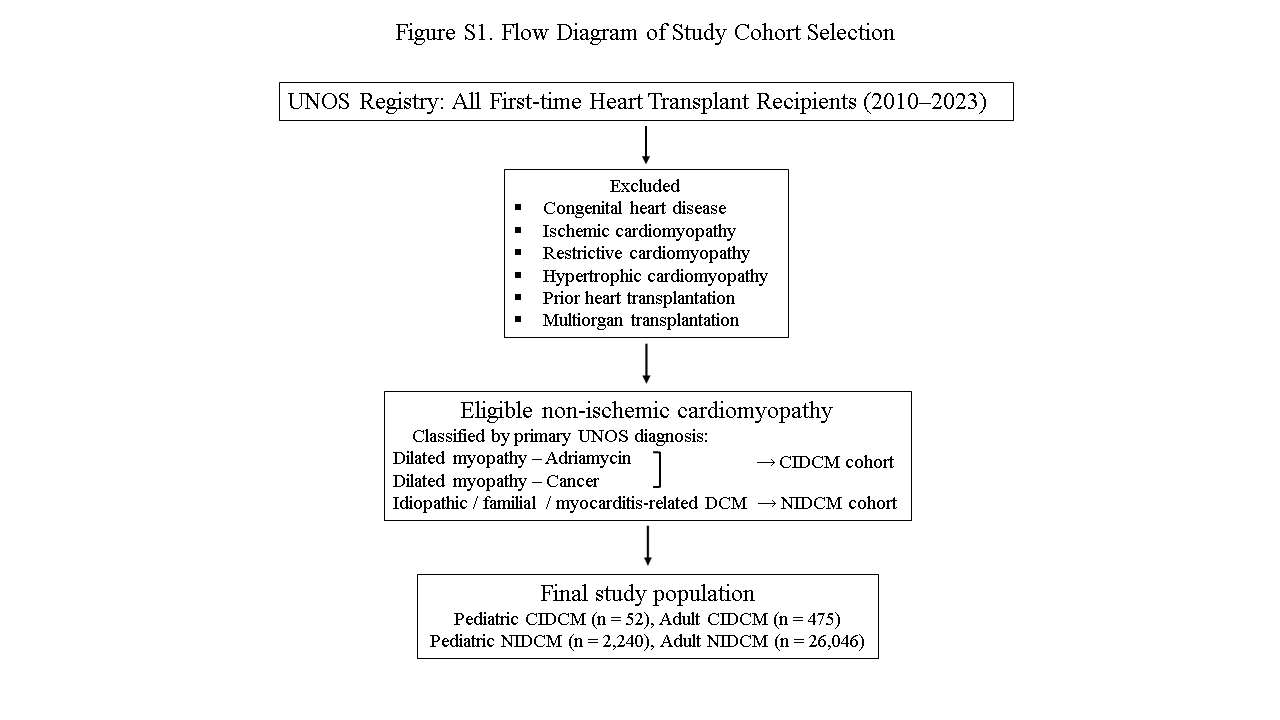


(Flow diagram of study cohort selection from the UNOS registry. Consecutive first-time heart transplant recipients were screened, excluded based on predefined criteria, and classified into chemotherapy-induced dilated cardiomyopathy (CIDCM) and non-ischemic dilated cardiomyopathy (NIDCM) cohorts according to primary listing diagnosis.)
